# Influence of Anesthetic Regimes on Extracellular Vesicles following Remote Ischemic Preconditioning in Coronary Artery Disease

**DOI:** 10.3390/ijms25179304

**Published:** 2024-08-28

**Authors:** Phuong N. V. Pham, Loubna Yahsaly, Crista Ochsenfarth, Bernd Giebel, Romina Schnitzler, Peter Zahn, Ulrich H. Frey

**Affiliations:** 1 Department of Anesthesiology, Intensive Care, Pain and Palliative Care, Marien Hospital Herne, Ruhr-University Bochum, 44801 Bochum, Germany; phuong.pham@ruhr-uni-bochum.de (P.N.V.P.); crista.ochsenfarth@elisabethgruppe.de (C.O.); 2 Department of Cardiology, University Hospital Essen, University of Duisburg-Essen, 45147 Essen, Germany; loubna.yahsaly@uk-essen.de; 3 Institute for Transfusion Medicine, University Hospital Essen, University of Duisburg-Essen, 45147 Essen, Germany; bernd.giebel@uk-essen.de; 4 Department of Anesthesiology, Intensive Care and Pain Medicine, BG University Hospital Bergmannsheil, Ruhr-University Bochum, 44789 Bochum, Germany; romina.schnitzler@ruhr-uni-bochum.de (R.S.); peter.zahn@ruhr-uni-bochum.de (P.Z.)

**Keywords:** RIPC, remote ischemic preconditioning, extracellular vesicles, micro-RNA, cardioprotection, anesthetics, propofol

## Abstract

Remote ischemic preconditioning (RIPC) reduces ischemia-reperfusion injury in aortocoronary bypass surgery, potentially via extracellular vesicles (EVs) and their micro-RNA content. Clinical data implicate that propofol might inhibit the cardioprotective RIPC effect. This prospective, randomized study investigated the influence of different anesthetic regimes on RIPC efficacy and EV micro-RNA signatures. We also assessed the impact of propofol on cell protection after hypoxic conditioning and EV-mediated RIPC in vitro. H9c2 rat cardiomyoblasts were subjected to hypoxia, with or without propofol, and subsequent simulated ischemia-reperfusion injury. Apoptosis was measured by flow cytometry. Blood samples of 64 patients receiving anesthetic maintenance with propofol or isoflurane, along with RIPC or sham procedures, were analyzed, and EVs were enriched using a polymer-based method. Propofol administration corresponded with increased Troponin T levels (4669 ± 435.6 pg/mL), suggesting an inhibition of the cardioprotective RIPC effect. RIPC leads to a notable rise in miR-21 concentrations in the group receiving propofol anesthesia (fold change 7.22 ± 6.6). In vitro experiments showed that apoptosis reduction was compromised with propofol and only occurred in an EV-enriched preconditioning medium, not in an EV-depleted medium. Our study could clinically and experimentally confirm propofol inhibition of RIPC protection. Increased miR-21 expression could provide evidence for a possible inhibitory mechanism.

## 1. Introduction

Acute myocardial infarction is one of the leading causes of morbidity and mortality worldwide. Impaired coronary perfusion during myocardial ischemia leads to significant hypoxia-related damage to the myocardium. In this context, rapid restoration of coronary blood flow is essential for maintaining left ventricular ejection fraction and preventing ventricular remodeling events [1]. However, while timely reperfusion is necessary to supply ischemic areas, reperfusion simultaneously causes partially irreversible damage [2]. The damage following successful revascularization is termed ischemia/reperfusion (I/R) damage (Ischemia-Reperfusion-Injury [IRI]) and is considered to be a consequence of calcium overload, oxidative stress, and inflammation [1,3]. IR damage is mainly observed after percutaneous coronary intervention (PCI), thrombolysis, or coronary artery bypass graft (CABG) surgery.

An endogenous approach to protection from IRI is ischemic preconditioning. Several studies have shown a protective effect of preconditioning, with reduced infarct size as the primary endpoint [1]. The principle of ischemic preconditioning offers an innovative therapeutic option to protect the heart from IR damage and to improve outcomes after myocardial infarction, acute coronary syndrome, and cardiac surgery. The idea of remote ischemic preconditioning (RIPC) describes the performance of episodes of ischemia and reperfusion on organs remote from the heart or on ischemia-robust body parts (e.g., the forearm) [4]. Hausenloy et al. applied the methodology of RIPC using a blood pressure cuff, which had already been tested in animal-experimental studies, to patients undergoing elective CABG surgery. They induced ischemia in the upper limb using a blood pressure cuff and could show a 43% postoperative lower serum troponin T concentration in the RIPC group compared to the control group [3].

However, two large multicenter trials failed to demonstrate the protective effects of remote ischemic preconditioning [5,6]. Based on the contradictory study results, propofol was assumed to be one confounding factor since propofol was administered in 90 to 100% of the latter multicenter studies. In contrast, a clinical study showed a protective effect only when isoflurane was used as an anesthetic [7]. This effect was confirmed in a study in rats [8]. Moreover, another study proposed that propofol impairs the release or transport of humoral factors to the myocardium [9], whereas the inhibitory effect of propofol was shown to be dependent on the time of application [10]. The underlying mechanism by which cardioprotection is mediated and possibly inhibited by propofol remained an open question. In addition to humoral factors such as adenosine, bradykinin, or nitric oxide, neuronal and systemic factors in the intercellular signal transduction of RIPC might play a major role [11,12]. In this context, extracellular vesicles (EVs) play a role as possible intercellular transporters of the RIPC effect.

EVs are a heterogeneous group of membranous nanoparticles that can be secreted by almost all cell types. As mediators of intercellular communication, they can deliver their contents, such as micro-RNA (miRNA), proteins, and lipids, to target cells, thereby modulating the recipient cell phenotype [13]. Depending on their biogenesis and size, exosomes (50 to 150 nm), microvesicles (100 to 1000 nm), and apoptotic bodies (up to 5 µm), as well as some minor EV groups, are currently distinguished. The role of EVs in pathophysiological and physiological cellular processes has attracted much research interest in the last decade. Their quantity, loading, and origin vary depending on the underlying pathophysiological process [14]. In cardiovascular disease, EVs play a role in developing atherosclerosis, inflammation, and cardiac fibrosis [15]. Moreover, EVs were shown to be involved in mediating the RIPC effect [16,17,18,19]. However, the isolation of plasma-derived EVs is particularly challenging due to their similar size and density to other plasma components, such as lipoproteins [20,21], and different isolation methods may influence the results [22].

Regarding the EV cargo, miRNAs have gained considerable attention in recent years. For example, EV-derived miRNAs are present in different amounts depending on the underlying cardiovascular disease, such as coronary artery disease or heart failure [15]. In addition, the EV miRNA signature may also be altered in cardioprotective or repair processes, such as RIPC [23,24]. To our knowledge, the role of propofol in inhibiting the efficacy of RIPC and its influence on the microRNA signature of EVs have not yet been extensively analyzed in clinical settings. Therefore, we tested the following hypotheses using a translational approach:

1. Ischemic preconditioning performed in vitro leads to cell protection, while the protective effect is EV-mediated, and using the anesthetic propofol reverses this protective effect.
2. Usage of propofol for anesthetic maintenance results in an inhibition of the cardioprotective RIPC effect by signs of different postoperative troponin T concentrations.
3. In the setting of CABG surgery, decreased expression of cardioprotective miRNAs occurs after RIPC in coronary patients when anesthesia is maintained using propofol.

Given the lack of standardized protocols, this research further compares different enrichment methods to determine their efficacy in quantifying miRNA from EVs. Selecting the most appropriate method is a critical aspect of the study design and should be based on the research’s specific objectives or intended applications. Consequently, we designed our experiments to identify the optimal enrichment technique for miRNA quantification from EVs.

## 2. Results

### 2.1. Evaluation of Different EV Enrichment Methods

In order to determine the most suitable EV enrichment method for miRNA analysis from either cell culture or patient plasma, we evaluated plasma samples from four patients participating in the clinical study prior to induction of anesthesia. We assessed particle concentration and the ratio of nanoparticle to protein concentration to estimate the purity of EV preparations (Purity Index). Further, the average particle size was measured to ensure alignment with the predefined region of interest (ROI) of 50 to 150 nm.

**Figure 1:**
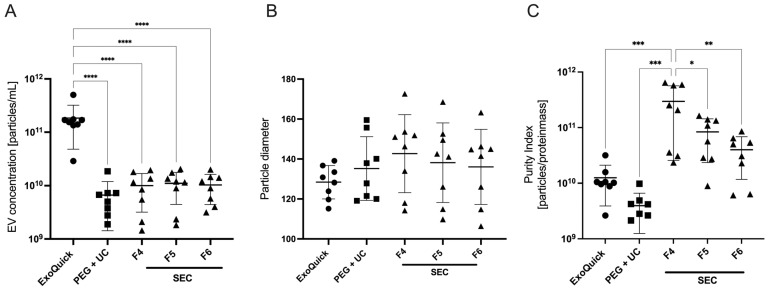
Mean measured EV concentration (**A**), particle diameter (**B**), and purity index (**C**) of three EV enrichment protocols (abbreviations: PEG + UC: Polyethylene glycol precipitation followed by ultracentrifugation, SEC F4–F6: Size exclusion chromatography, fractions 4–6), plasma of four patients prior to anesthesia induction, Mean ± SD; **** *p* = 0.0001, *** *p* = 0.0004, ** *p* = 0.002, * *p* = 0.014.

The results showed a significantly higher particle concentration of 1.8 × 10^11^ ± 1.3 × 10^11^/mL after applying the ExoQuick protocol. This concentration was significantly higher compared to those achieved using either PEG + UC or SEC protocols, as determined by a one-way ANOVA followed by Dunnett’s post hoc test (Figure 1A). Analysis of the Purity Index demonstrated a higher index in SEC samples, with the highest index in the fourth fraction (3 × 10^11^ ± 2.7 × 10^11^) as determined by one-way ANOVA followed by Tukey’s post hoc test, while it was 1.3 × 10^10^ ± 8.6 × 10^9^ for ExoQuick and 3.9 × 10^9^ ± 2.7 × 10^9^ for PEG + UC (Figure 1C).

In terms of particle diameters, the EVs enriched with ExoQuick showed the lowest size scatter. With a diameter of 128 ± 8.3 nm, they were closest to the reference interval of 50 to 150 nm, which corresponds to the size of exosomes. However, no statistical significance was shown here (Figure 1B).

**Figure 2:**
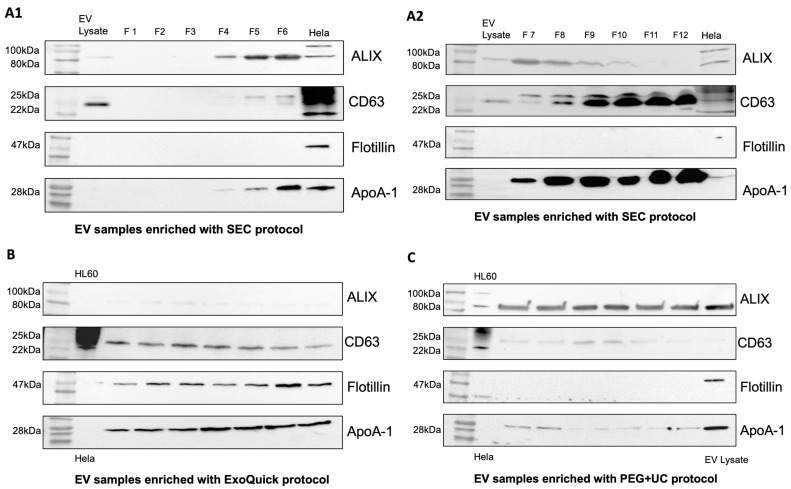
Western Blot. Only EV samples enriched with ExoQuick (**B**) show all tested EV characteristic proteins ALIX, Flotillin-1, and CD63 in comparison to samples enriched with the SEC (**A1**,**A2**) and PEG + UC (**C**) protocols. Comparison of contamination between EV-enrichment protocols by testing for ApoA-1. SEC: moderate contamination with ApoA-1 in earlier fractions, ALIX increasingly detectable in earlier fractions, CD63 in later fractions (**A1**,**A2**), ExoQuick: weak detection of ALIX, clear detection of CD63, Flotillin, and ApoA-1 (**B**), PEG + UC: clear detection of ALIX, weak detection of CD63 and ApoA-1, Flotillin undetectable (**C**).

To confirm the presence of EVs in the samples, Western blots were performed using EV-associated proteins CD63, Flotillin-1, ALIX, and apolipoprotein A-1 (ApoA-1) as a contamination marker. EV samples enriched using the SEC protocol showed moderate contamination with ApoA-1 in fractions 4–6 (Figure 2(A1,A2)). This contamination increased steadily in subsequent fractions 7–12. In addition, ALIX was increasingly detectable in the earlier fractions, and CD63 was increasingly detectable in the later fractions. Compared with the ExoQuick protocol, Flotillin-1 was not detectable in the SEC samples (Figure 2(A1,A2)). EV preparations obtained by the ExoQuick protocol showed a weak presence of ALIX, whereas CD63 and Flotillin-1 were clearly detectable. ApoA-1 appeared as a distinct band in the Western blot, indicating contamination with lipoproteins (Figure 2B). In samples obtained by applying the PEG + UC protocol, ALIX was clearly visualized, whereas CD63 was weakly detectable and flotillin-1 was undetectable (Figure 2C).

**Figure 3:**
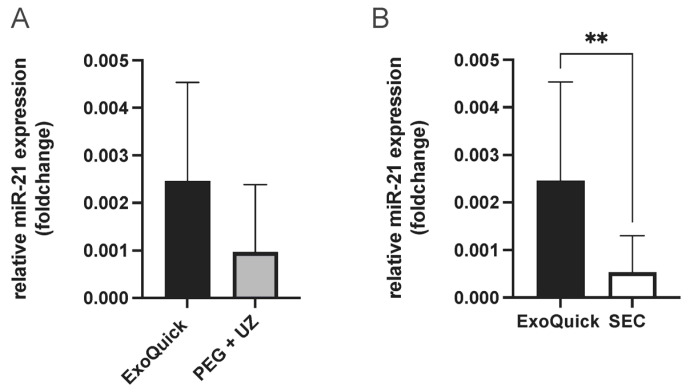
Evaluation of EV enrichment protocols (ExoQuick vs. PEG + UC (**A**) and ExoQuick vs. SEC, combined fractions 4–6 (**B**), comparison of relative miR-21 expression (foldchange), highest miR-21 concentration with samples enriched using the ExoQuick protocol, Statistics: Mean ± SD; unpaired *t*-test, ** *p* = 0.004.

To determine the best enrichment method for analyzing miRNAs present in extracellular vesicles (EVs), miR-21 was quantified from EVs. MiR-21 was selected due to its association with cardioprotection in the context of RIPC [23]. This resulted in the highest miR-21 concentration from EV samples enriched using the ExoQuick protocol. While the comparison with samples enriched with PEG + UC protocol showed no statistical significance, a significantly higher miR-21 concentration was shown when compared to SEC samples (unpaired *t*-test, *p* = 0.004, Figure 3). Therefore, this protocol was used for the following experiments to obtain the most comprehensive results possible.

### 2.2. Effect of Hypoxic Preconditioning on H9c2 Cells

**Figure 4:**
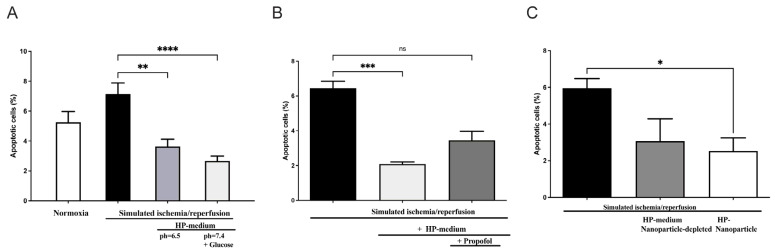
Effect of preconditioning medium (Hypoxic preconditioning in vitro) on apoptosis of H9c2 rat cardiomyoblast: (**A**) H9c2 cells were incubated in hypoxic preconditioning medium (HP medium) before being exposed to simulated myocardial ischemia-reperfusion injury (SIR) by 18 h of hypoxia followed by 6 h of reperfusion, which resulted in significant apoptosis reduction (n = 11, statistics: Kruskal–Wallis tests followed by Dunn’s post-hoc test (for multiple comparisons), **** *p* ≤ 0.0001, ** *p* ≤ 0.001). (**B**) Propofol addition inhibits apoptosis reduction in H9c2 cells incubated with HP medium. (n = 5; statistics: Kruskal–Wallis tests followed by Dunn’s post-hoc test for multiple comparisons, *** *p* ≤ 0.001, ns = *p* > 0.05). (**C**) EVs isolated from a preconditioning medium reduce apoptosis induced by SIR, whereas EV depletion in the HP medium has no protective effect. (n = 3, statistics: unpaired *t*-test, * *p* ≤ 0.05). Results are presented as mean ± SEM.

In order to investigate the role of humoral factors, especially EVs, on cardiomyocytes with the potential inhibitory effects of propofol, we used the model of hypoxic preconditioning (HP) in cardiomyoblasts (H9c2 cells). For this purpose, recipient H9c2 cells were treated with an HP medium and subsequently exposed to a simulated ischemia/reperfusion (Supplementary Figure S3). Apoptosis was measured after 18 h of hypoxia using flow cytometry with fluorescent staining. In contrast to continuous normoxia under serum deprivation (apoptosis: 5.25 ± 0.7%), apoptosis increased to 7.15 ± 0.7% after hypoxia and reperfusion (Figure 4A).

However, cells cultured in preconditioning medium (pH = 7.4 + glucose) showed a statistically significant decrease in apoptosis upon subsequent hypoxia and reoxygenation (H/R control: 7.15 ± 0.7% versus HP medium + H/R: 2.67 ± 0.3%; *p* < 0.0001, n = 11). In order to confirm the effect of the preconditioning medium under glucose deprivation and a pH of 6.5, simulated ischemia/reperfusion was also performed in an HP medium (pH = 6.5 without glucose). Culturing cells in HP medium prior to subsequent hypoxia and reoxygenation also resulted in significantly reduced apoptosis, though the statistical significance was slightly lower with *p* < 0.001 (Figure 4A). Thus, the preconditioned humoral factors alone result in cell protection from hypoxia, as demonstrated under glucose deprivation and a lower pH of 6.5.

We next investigated the role of propofol on cellular protection when the preconditioning medium was supplemented with propofol incubation. Here, the apoptosis-reducing effect was again demonstrated by incubating the cells in the preconditioning medium alone (*p* = 0.0004), while this apoptosis-reducing effect was diminished when incubation of the preconditioning medium was performed in the presence of propofol (Figure 4B).

To investigate whether EVs are responsible for the protective effect, we conducted simulated ischemia/reperfusion experiments using EV-depleted and EV-enriched media. As shown in Figure 4C, a significantly reduced apoptosis rate was demonstrated in cells subjected to simulated ischemia/reperfusion previously incubated with EVs. Interestingly, no significant decrease in apoptosis rate was seen upon incubation with EV-depleted preconditioning medium in cells subsequently subjected to simulated ischemia/reperfusion (Figure 4C). These results suggest a cardioprotective effect of the hypoxic preconditioned medium by using preconditioning EVs, but not in cells that were cultured with EV-depleted preconditioning medium. Thus, EV depletion appears to attenuate the cell-protective effect of HP. This provides evidence for possible EV-mediated cell protection during hypoxic preconditioning of H9c2 cells performed in vitro.

### 2.3. Clinical Study on the Role of EVs and Propofol in RIPC

We next investigated whether the in vitro results translate in vivo in a prospective randomized trial in CABG patients. Table 1 shows the demographic data of the four randomized groups. There were no significant differences between the groups in terms of demographics and clinical characteristics.

**Figure 5:**
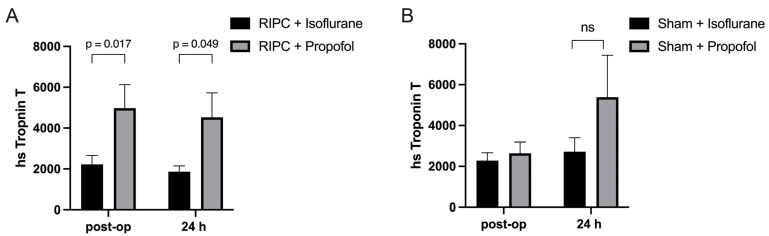
Myocardial damage is higher under propofol anesthesia: Troponin-T levels immediately after operation and 24 h post-surgery. Significantly higher troponin-T concentrations were measured in patients who received RIPC under propofol anesthesia in comparison to those under isoflurane anesthesia (**A**), with no statistical significance between isoflurane and propofol sham groups (**B**). Statistics: Mean ± SEM, *p* = 0.017 and 0.049, ns = *p* > 0.05, unpaired *t*-test.

In this clinical study, the concentration of Troponin-T, a biomarker indicative of myocardial damage, was measured in coronary artery bypass graft (CABG) patients to assess the impact of RIPC when administered in conjunction with either propofol or isoflurane. Blood samples were collected from the subjects both immediately after the operation and again 24 h post-surgery to monitor changes in the Troponin-T concentration over time. Specifically, the group that received RIPC while maintained under propofol anesthesia, demonstrated substantially higher Troponin-T levels (4669 ± 435.3 pg/mL) (Figure 5A). In contrast, the Troponin-T levels in the group receiving RIPC under isoflurane anesthesia were significantly lower (2044 ± 258.7 pg/mL) (Figure 5A). This result suggests that propofol anesthesia may interfere with the protective effects of RIPC against myocardial injury, as indicated by significantly higher Troponin-T concentrations observed in the propofol group compared to those patients maintained under isoflurane anesthesia. No statistical significance could be shown between the isoflurane and propofol sham groups, though a higher Troponin-T concentration could be seen in the propofol group 24 h post-operatively (Figure 5B).

### 2.4. Particle Size and Concentration

**Figure 6:**
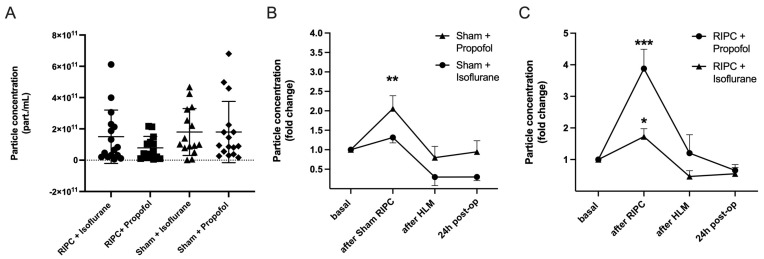
RIPC leads to the highest increase in particle concentration under propofol anesthesia. No significant differences in basal particle concentration between test groups (**A**) Mean ± SD, Analysis of variance, *p* = 0.27. Particle concentration throughout the operation (fold change), comparison of Sham Propofol vs. Isoflurane (**B**): an increase in particle concentration was found using propofol (** *p* = 0.0046). RIPC Propofol vs. Isoflurane (**C**): RIPC leads to an increase in particle concentration, which is highest with propofol (*** *p* = 0.0005) in comparison to isoflurane (* *p* = 0.014). Statistics: One sample *t*-test, Mean ± SEM.

Nanoparticle tracking analysis (NTA) was performed for the measurement of particle concentrations. For this purpose, particle concentrations were measured in the size range of 50 to 150 nm (ROI), corresponding to the expected exosome size, to differentiate EV subtypes approximately. The basal particle concentration was first quantified under control conditions. There were no significant differences between the groups, *p* = 0.26 (Figure 6A).

The particle concentration was then measured over the course of the operation. Blood samples were taken before RIPC after induction of anesthesia (basal), five minutes after remote ischemic preconditioning, after termination of the heart-lung machine (HLM), and 24 h post-operatively. All test groups showed an increase in the plasmatic EV concentration, whereby the increase was most pronounced in the patient groups following ischemic remote preconditioning. The highest rise in particle concentration could be shown in patients receiving RIPC under propofol anesthesia (foldchange (fc) = 3.88 ± 2.21, *** *p* = 0.0005; Figure 6C). Comparison of randomization groups in isoflurane patients revealed that the mean particle concentration initially increased significantly in the RIPC only (fc = 1.82 ± 0.97, * *p* = 0.01) and decreased until the end of surgery and the next day. The sham procedure did not lead to a significant rise in EV concentration in isoflurane patients, whereas in propofol patients, particle concentrations increased significantly (Figure 6B).

### 2.5. Detection of miRNA-21 Expression from EVs by Single Assay Analysis

**Figure 7:**
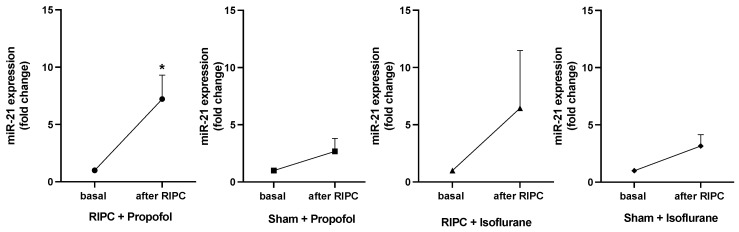
Significant rise in miR-21-5p concentration only under propofol anesthesia after RIPC. MiR-21-5p concentration basal and after RIPC maneuver. A rise in concentration could be shown in the RIPC isoflurane and propofol groups, whereas only in patients receiving RIPC under propofol anesthesia did the miR-21-5p concentration increase significantly (* *p* = 0.0157), statistics Mean ± SEM, one sample *t*-test.

Figure 7 shows the expression fold changes of miR-21-5p in all patients at baseline and after the RIPC maneuver. MiR-21-5p expression was analyzed due to its pivotal role in cardioprotection in the context of preconditioning, as previously shown [23,25]. In both RIPC groups, isoflurane and propofol, a higher miR-21-5p concentration was measured than in the Sham groups. Only the group receiving RIPC under propofol anesthesia showed a significant increase in miR-21-5p concentration (*p* = 0.0157). In contrast to the results by Lassen et al., analysis of miR-144-3p, miR-16-5p, and miR-451a showed no significant expression fold changes in any study group [19].

## 3. Discussion

Cardiovascular diseases remain a significant cause of morbidity and mortality in modern society. Remote ischemic preconditioning (RIPC) has emerged as a promising strategy to attenuate ischemia-reperfusion injury (IRI) in cardiac surgery patients. While EVs have been identified as humoral factors in intercellular communication in both physiological and pathophysiological processes, the exact mechanism of mediating cardioprotection and its possible inhibition still remain largely unclear. Possibly, a transfer of EV cargo, such as miRNA, achieves an alteration of gene expression and phenotypical changes in the recipient cell [13]. While experimental studies propose a cardioprotective effect in vitro and in vivo [18,19,26], clinical studies failed to conclusively affirm RIPC’s protective effect [5,6], raising the question of a potential inhibition by propofol.

Therefore, we explored the role of EVs in mediating cardioprotection in vitro. Our results demonstrate that cardioprotective effects are only evident in the presence of EVs, underlining the critical role of EV mediation. We further investigated the effect of anesthesia maintenance with either propofol or isoflurane on RIPC’s cardioprotection. Our data reveal increased myocardial injury associated with propofol anesthesia despite RIPC, as evidenced by significantly elevated Troponin-T levels. A substantial rise in miR-21 concentration among patients receiving RIPC under propofol anesthesia further suggests that propofol may interfere with RIPC on an intercellular level.

### 3.1. EVs in Hypoxic Preconditioning Medium and Plasma as Mediators of Cardioprotection

Our experiments could successfully reproduce the protective effect of ischemic preconditioning within an in vitro H9c2 cell model. We were able to demonstrate a protective, apoptosis-reducing effect by >60% when employing three cycles of 5-min H/R before 18 h of ischemia and 6 h of reperfusion. This observation aligns with the results by Mudaliar et al., who reported a similar attenuation of apoptosis in recipient cells [26]. In 2018, Chen et al. confirmed a protective effect of hypoxic preconditioning in H9c2 cells, which were then subjected to palmitic acid-induced lipotoxicity following a brief hypoxia exposure of 5 min to 2 h [27]. Using the same principle, Hu et al. also confirmed reduced apoptosis through hypoxic preconditioning of H9c2 cells [28]. A recent in vitro study by Yan et al. explored possible mediators of cell protection and demonstrated that the apoptosis-reducing effect of HP on mouse cardiomyoblasts was associated with alterations in the miRNA signature of C2C12 cells and the secreted EVs, including a notable upregulation of miR-182-5p in HR-EVs, which is associated with an enhancement of angiogenesis and neuroprotection [29].

After showing a protective effect against stress-induced apoptosis by exposure to preconditioning HP medium, we further explored the hypothesis of EV-mediated protection by assessing the impact of EV depletion. The protective effect observed after preconditioning and incubating the recipient cells with EVs was absent in the EV-depleted HP medium. Our findings, therefore, suggest EV-mediated protection. In a previous study, we already demonstrated that serum from preconditioned patients showed an increase in EV nanoparticle concentration, and subsequent incubation of H9c2 cells with these EVs resulted in a reduction of hypoxia-induced apoptosis [30]. Similarly, Li et al. observed that EVs from preconditioned rats not only reduced apoptosis in H9c2 cells but also improved cardiac function and decreased infarct size in vivo [31].

Giricz et al. further confirm the significance of EVs for cardioprotection, as hearts perfused with EV-depleted plasma exhibited no myocardial protection post-RIPC, whereas perfusion with EV-enriched plasma achieved a cardioprotective effect [16]. Vicencio et al. showed a cross-species effect of RIPC by transferring EVs enriched from healthy subjects post-RIPC to cardiomyocytes [18]. The results of Lassen et al. further underline the feasibility of transferring RIPC by perfusing rat hearts with EVs, which were enriched from the plasma of healthy human subjects after receiving RIPC [19]. The transmission of the RIPC signal by EVs might be associated with EV concentration [18,23] and may occur through direct receptor-ligand interaction [18] or through vesicle fusion with the target cell membrane, thereby transferring miRNA [19]. Our experiments underline the association between EV concentration and cardioprotection by showing an increase in nanoparticle concentration after RIPC compared to Sham.

### 3.2. Propofol as an Inhibitory Factor of Remote Ischemic Preconditioning

The HP-mediated apoptosis reduction could be abolished in our in vitro cell model by simultaneously cultivating the H9c2 cells with propofol and the preconditioning medium. Consistent with these in vitro findings, we could demonstrate that propofol anesthesia inhibits cardioprotection, showing significantly elevated Troponin-T concentrations as a sign of higher myocardial damage in patients who underwent propofol anesthesia despite receiving RIPC.

Similar observations have already been made by Kottenberg et al., who have previously shown a loss of cardioprotection in CABG patients undergoing propofol anesthesia, while those who had isoflurane administration still maintained the RIPC effect [7]. The group attributed the reduction of RIPC-mediated cardioprotection to the absence of STAT5 pathway activation on a cellular level [32]. These findings gain clinical significance in light of Phase III trials, namely the ERICCA [6] and the RIPHeart trials [5], which both failed to confirm the cardioprotective benefits of RIPC in cardiac surgeries. Notably, propofol was predominantly employed as an anesthetic agent [5,6]. Supporting propofol’s inhibitory effect, a meta-analysis of 55 randomized studies revealed a lower mortality rate in cardiac surgery patients when volatile anesthetics were administered in combination with RIPC, as opposed to propofol-based total intravenous anesthesia [33]. In the past, the cardioprotective effects of volatile anesthetics have been discussed [34], suggesting a possible synergy with RIPC. Lower troponin T levels in the isoflurane group 24 h post-operatively without RIPC might support a potential cardioprotective effect of volatile anesthetics and the inhibitory effects of propofol.

The mechanism behind this phenomenon is currently discussed, with potential factors including intracellular signal inhibition [32], the reduced release of humoral factors [35], or impaired uptake in the presence of propofol.

Bunte et al. demonstrated that plasma from rats receiving RIPC under pentobarbital anesthesia protected naive rat hearts against global myocardial I/R injury. This effect was not observed under propofol anesthesia and thus indicated an inhibition by propofol at an intercellular level [9]. Our in vitro and clinical findings underline the inhibitory impact of propofol. As we have shown the feasibility of transferring a hypoxic preconditioning medium for the cell protection of untreated cells, a humoral mediation of cardioprotection can be assumed. The absence of protective effects after EV depletion or under-propofol exposure suggests EVs as mediators on the one hand and interference of propofol in the EVs-mediated signal transfer on the other hand. Furthermore, the results of our clinical study showed an increase in particle concentration in both RIPC intervention groups 5 min after receiving the RIPC maneuver while only having cardioprotective effects in the group under isoflurane administration.

These findings further corroborate the interference of propofol on an intercellular level, possibly mitigating transport or signal transduction to the recipient cell but not the release of EVs.

It is essential to note that the propofol used in our study, commonly used in clinical settings, consists of a soy fat emulsion. Another aspect worth considering is a potential interaction between the soy fat emulsion and the lipid bilayer of the EVs, which might hamper the EV effect. On the other hand, Deng et al. also reported a decrease in vesicle secretion from endothelial cells during hypoxia/reoxygenation (H/R) when using a pure, fat-free propofol form [35], so lipid interaction might not be the sole interference mechanism.

Although our results indicate that propofol inhibits RIPC efficacy, alternative inhibitory mechanisms, and potential confounding factors may also play a role. For instance, Torregroza et al. demonstrated in vivo that RIPC does not confer cardioprotection in type 1 diabetes mellitus and hyperglycemic myocardium [36]. Jensen et al. showed that the release of cardioprotective humoral factors by RIPC is dependent on an intact afferent nerve system [37]. Given the complexity of comorbidities in patients with coronary artery disease, it is possible that propofol is not the only factor responsible for the observed inhibition.

### 3.3. Influence of Propofol on EV miRNA Signature

MiRNAs, contained in EVs and selectively transported and released to target cells, have been suggested as potential signaling pathways for remote ischemic preconditioning [24]. Lassen et al. detected elevated expressions of miR-16-5p, miR-144-3p, and miR-451a in EV preparations after RIPC and concluded that upregulation of these miRNAs leads to cardioprotection via the mTOR signaling pathway [19]. However, in our patient cohort, we could not demonstrate significant expression differences for miR-16-5p, miR-144-3p, or miR-451a, thus we could not confirm this assumption.

In our group’s previous research, the miRNA signature in the serum of patients after RIPC was analyzed, and a significantly increased concentration of miR-21 was associated with cardioprotection [23]. In vivo studies could show an upregulation of miR-21 in the context of ischemic conditioning [25,38]. At the same time, a knockdown abolished cardioprotection in rat hearts [25], thus underlining the potential role of miR-21 in mediating the IPC effect. Specifically, cardioprotection by miR-21 upregulation might involve the inhibition of pro-apoptotic genes, such as PDCD4 [25] or PTEN [39], Heat Shock Protein 70 (HSP70), as well as endothelial Nitric Oxide Synthase (eNOS) [40]. Our miRNA analysis shows an increase in miR-21 expression in RIPC groups under isoflurane and propofol anesthesia, while there was no significant increase in Sham groups.

Interestingly, the increase of miR-21 was most pronounced and significant in the RIPC group receiving propofol for anesthesia maintenance. Therefore, the hypothesis that the propofol-induced inhibition of RIPC cardioprotection is associated with a reduced expression of cardioprotective miRNAs, such as miR-21, cannot be confirmed. Our findings show, on the contrary, an increase in cardioprotective miR-21 despite showing an inhibition of cardioprotection. This observation is of outstanding interest since our clinical study could confirm diminished cardioprotection under propofol anesthesia by increasing Troponin T concentrations in patient plasma. Our data point to an inhibitory effect on the intercellular level in propofol patients, although the exact mechanism and miRNA-signaling pathway remain an open question. This assumption is in line with our in vitro studies proposing a possible rationale for increased miR-21 expression after RIPC despite propofol administration as an interference of RIPC mediation. Although RIPC leads to elevated EVs and cardioprotective miR-21 concentrations, propofol might impair the transfer and impact of humoral factors on cardiomyocytes [9].

### 3.4. Limitations

Although demographic data and clinical characteristics showed no significant differences between the intervention groups, there was a variation in EuroScore II values that did not reach statistical significance. Troponin-T concentrations were measured at two-time points, revealing significant differences. While the highest changes of Troponin-T are detected on the first postoperative day, we could not comment on total troponin release over 72 h, as performed in previous studies [7,23].

While inhibition on an intercellular level can be concluded from our study results, we did not investigate the potential direct influence of EVs on the pro-apoptotic effect of propofol in vitro. Further research would be valuable to understand the exact inhibitory mechanisms by which propofol inhibits RIPC efficacy. Such studies could include in vitro experiments comparing the influence of HP medium with or without EVs on H9c2 cells cultivated with propofol and exposed to SIR.

Nanoparticle tracking analysis does not allow differentiation of EVs from other similarly sized particles, such as very-low-density lipoproteins and chylomicron remnants (80–150 nm) [41]. Although EV release can be concluded by a relative rise in concentration after RIPC regardless of the anesthetic regimen, we registered higher particle concentrations in the Sham group when propofol was used as the anesthetic agent. This may originate from a temporary rise in lipid concentration in the patient blood caused by continuous propofol administration.

Finally, all intervention groups were induced to use propofol, which may have reduced the extent of myocardial protection and hampered the differences between the intervention groups (propofol groups vs. isoflurane groups).

## 4. Materials and Methods

### 4.1. Cell Culture Conditions

H9c2 rat cardiomyoblasts (obtained from the European Collection of Authenticated Cell Cultures [ECACC], Salisbury, UK) were maintained in Dulbecco’s Modified Eagle’s Medium (DMEM medium + GlutaMAX + 4.5 g/L D-Glucose, Waltham, MA, USA), supplemented with 10% fetal calf serum (FCS) in a 5% CO_2_ atmosphere at 37 °C. The cells were cultivated in cell culture bottles and split at a 1:3 or 1:4 ratio when the confluence stage (70–80%) was reached.

### 4.2. Different Medium Conditions

Dulbecco’s Modified Eagle Medium (DMEM), high glucose content, GlutaMAX™ Supplement, Thermo Fisher Scientific (Waltham, MA, USA) for H9c2-Cell culture medium was used for seeding donor cells. DMEM, high glucose content, GlutaMAX™ Supplement medium, and Thermo Fisher Scientific (Waltham, MA, USA) were used for seeding the recipient cells. DMEM, (+4 mM L-Glutamin) medium (pH = 6.5), Thermo Scientific (Waltham, MA, USA) +10 mM Hepes sodium salt (Sigma, Tokyo, Japan) was used for hypoxia experiments. DMEM (+4 mM L-Glutamin; +10 mM Hepes sodium salt (Sigma); +10 mM Glucose (Sigma; pH 7.4; Thermo Scientific, Waltham, MA, USA) was used for experiments during normoxia.

### 4.3. Hypoxic Preconditioning on H9c2-Cells

The ischemic preconditioning on H9c2 (donor) cells was accomplished as hypoxic preconditioning (HP) in three cycles of alternating 5-min hypoxia and reoxygenation. Then, these preconditioned media were added to recipient H9c2-cells, which were subjected to 18 h of hypoxia followed by 6 h of normoxia to simulate myocardial ischemia/reperfusion (SIR) injury (Supplementary Figure S3). To obtain the conditions of hypoxia/normoxia, cells were transferred into Billups-Rothenberg chambers (MIC-101, Billups-Rothenberg, Del Mar, CA, USA), followed by subsequent flushing of the chamber (5 L/min) with normoxic (5% CO_2_, 21% O_2_, balance nitrogen) or hypoxic (1% O_2_, 5% CO_2_, balance nitrogen; Air Liquide, Düsseldorf, Germany) carrier gas. These experiments were kept under serum deprivation under four different experimental conditions: (1) donor cells exposed to HP, (2) HP-medium recipient cells with subsequent SIR, (3) HP-medium recipient cells in the presence of propofol, with subsequent SIR, (3) normoxia control cells, and (4) SIR-control cells. H9c2 rat cardiomyoblasts were seeded in 6-well plates in DMEM medium (+GlutaMAX + 4.5 g/L Glucose) ±10% FCS at 37 °C for 24 h. The donor cell medium, which is subsequently subjected to hypoxic preconditioning (HP), was first renewed by fresh DMEM medium (+4 mM L-glutamine + 10 mM HEPES buffer ± 10 mM glucose, −FCS), while a glucose-rich medium (pH = 7.4), as well as the DMEM-medium without glucose (pH = 6.5), were used for the subsequent HP to investigate the effect of HP under the presence or absence of glucose and neutral pH conditions. During the HP, the remaining cell groups were incubated under normoxia (21% O_2_, 5% CO_2_). The HP medium was then transferred to the respective recipient cell group (±50 µm propofol) and incubated under normoxia for 6 h. Before transferring the HP medium to the corresponding recipient cell group, the donor cell media were transferred to conical centrifugal tubes, centrifuged for seven minutes at 450× *g* at 21 °C to remove dead cells and cell debris, and eventually transferred to the respective recipients.

In a later approach, recipient cells were either incubated with an EV-depleted HP medium or nanoparticles isolated from the HP medium. For the subsequent simulated ischemia/reperfusion, a normoxia control group was exposed to normoxia and maintained in the glucose-rich medium for 18 h, while the cell culture medium of cells, which received hypoxic treatment, was renewed by the use of the glucose-free medium (pH 6.5). These cells were then incubated for 18 h under hypoxia (1% O_2_, 5% CO_2_), followed by reperfusion, whereby the medium of all cells was renewed with a fresh glucose-rich medium (pH 7.4), and the cells were kept under a normoxic atmosphere for six hours. The primary endpoint was apoptosis, detected by flow cytometry.

### 4.4. Apoptosis Measurement Using Flow Cytometry

The cells were collected and stained according to an already established experimental protocol [30]: The medium of the respective cell groups was pipetted from the 6-well plates and transferred to centrifuge tubes, followed by the addition of 1 mL TrypLE™ followed by incubation for 8 min in TrypLE™ at 37 °C. At the end of the incubation time in the Tryp-LE, 1 mL of DMEM medium + 10% FCS was added to each well for inactivation. The TrypLE™ medium mixture was then added to the corresponding centrifuge tubes, and the cell suspension was centrifuged at 900× *g* and 4 °C for 5 min. The supernatant was aspirated, and the pellet was resuspended with 200 µL of Annexin V Binding Buffer. The target cell concentration was adjusted to 5 to 10 × 10^6^ cells/mL. The buffer-cell mixture was transferred to the respective flow cytometry tubes for subsequent staining. The cells were then stained by adding 5 µL FITC-Annexin-V and 5 µL 7-AAD. The stained cell suspension was vortexed for a few seconds and incubated for 15 min in the dark at room temperature. Subsequently, 4 mL of the diluted Annexin V Binding Buffer was added to each tube and centrifuged at 900× *g* and 4 °C for 5 min. The supernatant was discarded after centrifugation, and the pellet was resuspended in 200 µL of Annexin V Binding Buffer. After thorough vortexing, the cell-buffer mixture was placed on ice and measured immediately afterward. The Cytoflex S flow cytometer (Beckman Coulter) with CytExpert 2.3.0.84 software (Beckman Coulter, Brea, CA, USA) was used to measure apoptosis in the first series of experiments (hypoxic preconditioning in three cycles). For each sample, 20,000 cells were measured at a 60 µL/min flow rate.

### 4.5. Clinical Study Design

After approval of the ethics committee of the Ruhr University Bochum (Registration No.: 17-6290, 6 March 2018), 98 patients with a diagnosis of coronary artery disease were included in this prospective, randomized, and partially double-blind study (trial registration: DRKS00015682). The study comprised four groups, two of which received RIPC preoperatively after 30 min of anesthesia using either the volatile anesthetic isoflurane or propofol, while the other two groups received standard therapy (Sham) without RIPC. 64 patients completed the study protocol (see CONSORT diagram, Supplementary Figure S1). All patients underwent surgery between May 2019 and August 2020 at the Department of Thoracic and Cardiovascular Surgery at the University Hospital Bergmannsheil, Bochum, and were recruited by the Department of Anesthesiology. The following inclusion criteria were applied: coronary artery disease scheduled for isolated CABG with a heart-lung machine, age between 45 and 85 years, standard medication with statins and beta-blockers, as well as ASS present preoperatively. Intraoperatively, standard anesthesia with isoflurane/sufentanil or propofol/sufentanil and a standard intraoperative protocol (full heparinization with more than 400 s activated clotting time, protamine).

Exclusion criteria were as follows: acute myocardial infarction in the last 28 days, combined valvular vitium, unstable angina, dual antiplatelet therapy, or renal insufficiency with creatinine levels greater than 1.5 mg/dL. In addition, patients who had undergone emergency surgery required administration of phosphodiesterase inhibitors, or required installation of an intra-aortic balloon pump were post hoc excluded.

Induction of anesthesia was carried out according to the usual standards, with propofol as a hypnotic, sufentanil as an opioid, and rocuronium or pancuronium as non-depolarizing muscle relaxant. Anesthesia was maintained using a mixture of air/oxygen and isoflurane (MAC 0.8–1.0) or a continuous infusion with propofol of 5–8 mg/kg/h after induction of anesthesia. The RIPC procedure consisted of 3 cycles of 5-min suprasystolic blood pressure cuff inflations (>200 mmHg) on the left upper arm/5-min deflations, or Sham (cuff left uninflated for 30 min) after induction of anesthesia.

### 4.6. Sample Collection

Arterial blood samples were collected from patients 30 min after induction of anesthesia (basal), 5 and 60 min after RIPC (after RIPC), after discontinuation from the heart-lung machine (after HLM), and on the first postoperative day. Arterial blood was collected in 7.5 mL of EDTA and 8.2 mL of citrate monovettes (Sarstedt, Nürmbrecht, Germany). Subsequently, the monovettes were centrifuged at 2000× *g* for 10 min, the plasma supernatant was aliquoted to 500 µL each, and the reaction tubes were frozen at −80 °C until further use.

### 4.7. Enrichment of the Extracellular Vesicles

#### 4.7.1. Size Exclusion Chromatography (SEC)

Plasma samples were thawed on ice and centrifuged at room temperature at 1500× *g* for 15 min. Then, 500 µL of the supernatant was transferred to a clean reaction tube (DNA LoBind Tubes 1.5 mL, Eppendorf, Hamburg, Germany) and centrifuged at room temperature at 10,000× *g* for 10 min. Samples were passed through a 0.22 µm filter (Sarstedt, Nümbrecht, Germany) onto the separation columns (qEVoriginal 35 nm, IZON Science, Christchurch, New Zealand) and collected into clean reaction tubes in a total of 12 fractions of 500 µL each. Due to their shorter diffusion distance, EVs are thus trapped in earlier fractions (Supplementary Figure S2). The samples were then stored at −80 °C until further use.

#### 4.7.2. Polyethylene Glycol (PEG) Precipitation Followed by Ultracentrifugation

EV enrichment was done essentially as described elsewhere [42]. Briefly, plasma samples were thawed on ice, diluted with 500 µL NaCl 0.9%, and centrifuged at 10,000× *g* for 10 min at 4 °C. Supernatants were transferred to a clean reaction tube and incubated overnight at 4 °C for 16 h after the addition of 250 µL 50% *w*/*v* PEG 6000 (Sigma-Aldrich, Taufkirchen, Germany). Subsequently, precipitated EVs were pelleted by centrifugation at 1500× *g* and dissolved again, followed by ultracentrifugation at 19,500 RPM and 4 °C for 130 min (Optima L7-65 ultracentrifuge, rotor 50.4Ti, k-factor 252, Beckman Coulter, Brea, CA, USA). The resulting pellet was dissolved in 150 µL NaCl 0.9%, and the sample was stored at −80 °C until further use.

#### 4.7.3. Polymer-Based Enrichment Method

Plasma samples or HP-Medium thawed on ice were centrifuged (3000× *g*, 4 °C, 4 min), and the supernatant (400 µL) was transferred to a clean reaction tube (DNA LoBind Tubes 1.5 mL, Eppendorf, Hamburg, Germany). The sample was then centrifuged again (10,000× *g*, 4 °C, 15 min), and the supernatant was added to another clean reaction tube together with 400 µL HEPES/NaCl 1:100 and 200 µL ExoQuick™ solution (System Biosciences, CA, USA). After 18 h of incubation at 4 °C and centrifugation (1500× *g*, 4 °C, 30 min), EVs were dissolved with 170 µL HEPES/NaCl 1:100, followed by size exclusion chromatography using PD SpinTrap™ columns (Cytiva, Marlborough, MA, USA) filled with Sephadex™ G-25. The EV-enriched samples were used directly for further investigations or stored at −80 °C.

### 4.8. Western Blot Analysis

Western blot analyses were performed as described [23]. Briefly, 15 µg of protein were used in 4–20% precast gels (BioRad Laboratories Inc., Hercules, CA, USA) for both ExoQuick and PEG samples, after measuring the total protein concentration using a BCA protein assay (Thermo Fisher Scientific, Waltham, MA, USA). To visualize the protein increase over time as the recovered fractions progressed, 25 µL of each fraction from the SEC EV enrichment was added to each gel pocket. Positive controls were Hela whole cell lysate (sc-2200, Santa Cruz Biotechnology, Dallas, TX, USA), HL60 whole cell lysates (ab7914, Abcam, Cambridge, UK), and EV positive controls. Electrophoretic transfer from the gel onto a nitrocellulose membrane was done using a transfer cell (Trans-BlotR SD Semi-Dry Transfer Cell, Bio-Rad Laboratories, Hercules, CA, USA), and the successful protein transfer was validated by performing a reversible Ponceau S total protein staining. The following primary antibodies served as a qualitative validation of EV-associated proteins: ALIX Mouse monoclonal IgG_2a_ (C-11, sc-271975); Flotillin-1 mouse monoclonal IgG_1_ (C-2, sc-74566; all Santa Cruz Biotechnology, Inc., Dallas, TX, USA); and CD63 rabbit polyclonal IgG (ab68418, Abcam, Cambridge, UK). ApoA-1 mouse monoclonal IgG_2b_ (B-10, sc-376818, Santa Cruz Biotechnology, Inc., Dallas, TX, USA) was used as a contamination marker. The chemiluminescent reaction was detected in the Molecular Imager R ChemiDocTM XRS + Imaging System (Bio-Rad Laboratories GmbH, Munich, Germany).

### 4.9. EV Quantification

The Particle Tracking Analyzer NTA Zetaview^®^ (Particle Metrix GmbH, Meerbusch, Germany) was used for quantification as described previously [23]. The “region of interest” (ROI) was set to 50–150 nm [43] according to the expected size of exosomes.

### 4.10. Purity Index

In order to compare the purity index of the different EV enrichment methods, the particle concentration and protein mass ratio of the samples were calculated based on the lower protein content of EVs compared to lipoproteins, which consist mainly of membrane-forming lipids [44]. The protein concentration of all samples was measured using a microvolume spectrophotometer, and the particle concentration was measured using NTA as previously described. A higher value of the Purity Index indicates a higher proportion of EVs in the measured nanoparticles and thus indicates less contamination by lipoproteins in the sample. The Purity Index was calculated using the following formula:

$$Purity\;Index=\frac{particle\;concentration[{mL}^{-1}]}{protein\;mass[mg]}$$

### 4.11. Gene Expression Analyses

The mirVANA™ miRNA Isolation Kit (Invitrogen, Thermo Fisher Scientific, Waltham, MA, USA) was used to isolate miRNAs from the EVs. Diluted RNAse A (Thermo Fisher Scientific, Waltham, MA, USA) was added to the EVs to degrade RNAs outside of EVs and prevent possible contamination by non-EV-associated miRNAs. Cel-miR-39-3p (Invitrogen, Thermo Fisher Scientific, Waltham, MA, USA) was added as a reference to calibrate the samples (spike-in). Heparin was removed from samples using heparinase I (Sigma-Aldrich, Munich, Germany) as described [45]. MiRNAs were then transcribed into cDNAs using the TaqMan™ Advanced miRNA cDNA Synthesis Kit (Applied Biosystems, Waltham, MA, USA) using a poly-(A)-tail and universal random primers. Single quantitative real-time polymerase chain reaction was performed using the QuantStudio™ 7 Flex Real-Time PCR System (Applied Biosystems, Waltham, MA, USA) with the following assays: hsa-miR-144-3p (477913), hsa-miR-16-5p (477860), hsa-miR-21-5p (44975), hsa-miR-451a (478107), and cel-miR-39-3p (478293; all from Thermo Fisher Scientific Inc., Waltham, USA).

### 4.12. Statistics

Metric data were tested for normal distribution using Kolmogorov-Smirnov tests and reported as mean ± standard deviation in cases of normal distribution. Otherwise, the median and interquartile range (IQR) were reported. In the case of normal distribution, groups were tested using a two-tailed independent paired or unpaired *t*-test or analysis of variance (ANOVA); in the case of non-normally distributed variables, the Mann–Whitney U test or Kruskal–Wallis was used. Categorical variables were expressed as numbers and percentages and analyzed using the chi-square test. For a number of cases less than five per group, Fisher’s test was used. qPCR assays were evaluated using the delta-CT method [46], and relative miRNA expression was calculated using the spike-in cel-miR-39-3p as a control. The significance level was set at α = 0.05. All statistical analyses were carried out with the SPSS software (Version 26, IBM, Armonk, NY, USA) or GraphPad Prism 9 (GraphPad Software, San Diego, CA, USA).

## 5. Conclusions

In a translational approach, we could demonstrate an inhibition of hypoxic preconditioning in the presence of propofol in vitro and confirm these findings clinically by showing higher myocardial damage in CABG patients receiving RIPC under propofol anesthesia. Additionally, we could validate the role of EVs as mediators of preconditioning on an intercellular level by abolishing reduced apoptosis by EV depletion in vitro and demonstrating significantly increased nanoparticle concentrations after RIPC in plasma. A decreased concentration of cardioprotective miRNA, such as miR-21, under propofol exposure cannot be affirmed. Moreover, a sustained increased expression of cardioprotective miR-21 despite propofol administration might further underline a potential inhibition by propofol on an intercellular level. The combined results of our in vitro study and prospective randomized trial might serve as a basis for further studies investigating the mechanisms of RIPC inhibition by propofol. Future studies could investigate the influence of EVs on propofol’s inhibitory effects in vitro or examine the biophysical properties and characteristics of EVs in patients undergoing CABG surgery with either propofol-based or propofol-free anesthesia. Additionally, a follow-up beyond 24 h post-surgery and a larger patient cohort, either excluding known confounding comorbidities (such as diabetes mellitus) or specifically focusing on them, would also be of significant interest.

## Figures and Tables

**Table 1:** Demographic data and clinical characteristics. Categorical variables are presented as n (%), normally distributed continuous variables as mean ± (SD), and non-normally distributed continuous variables as median [IQR]; *p*-values refer to the Chi-square test, ANOVA, or Kruskal–Wallis test *.

	Total	RIPC Isoflurane	RIPC Propofol	Sham Isoflurane	Sham Propofol	*p* Value
Number (n, %)	64	16 (25)	14 (21.9)	17 (26.6)	17 (26.6)	
Age (years)	68.6 ± 8.2	71.4 ± 8.9	66.9 ± 6.8	69.8 ± 6.2	66.2 ± 9.8	0.237
Sex (male/female)	50/14	15/1	9/5	12/5	14/3	0.204
Vessels (n, %)						
2	8 (12)	4 (25)	0 (0)	2 (12)	2 (12)	
3	56 (88)	12 (75)	14 (100)	15 (88)	15 (88)	0.231
BMI (kg/m^2^)		25.9 [4.3]	26.4 [8.8]	27.7 [7.5]	26 [6.8]	0.517 *
EuroScore II (%)		1.94 [2.36]	1.03 [1.66]	1.15 [0.54]	1.85 [1.5]	0.089 *
Diabetes (n, %)	16 (25)	4 (25)	2 (14)	6 (35)	3 (18)	0.508
Smoking (n, %)						
currently	21 (33)	4 (25)	5 (36)	5 (29)	7 (41)	
formerly	9 (13)	2 (13)	1 (7)	2 (12)	3 (18)	0.764
Medication (n, %)						
ASA	56 (88)	14 (88)	10 (71)	15 (88)	17 (100)	0.125
Clopidogrel	5 (8)	1 (6)	2 (14)	0 (0)	2 (12)	0.444
Beta-blockers	48 (75)	14 (88)	9 (64)	12 (71)	13 (77)	0.496
Insulin	6 (10)	1 (6)	0 (0)	3 (19)	2 (13)	0.338
Bypass time (min)	95 ± 42	90 ± 45	100 ± 56	89 ± 28	104 ± 40	0.665
Aortic clamping time (min)	51 ± 32	49 ± 20	44 ± 38	54 ± 32	55 ± 37	0.767

## Data Availability

The data presented in this study are available on request from the corresponding author. The data are not publicly available due to privacy.

## Supplementary Materials

The following supporting information can be downloaded at: https://www.mdpi.com/article/10.3390/ijms25179304/s1.
