# Supplementary material for: Influence of Anesthetic Regimes on Extracellular Vesicles following Remote Ischemic Preconditioning in Coronary Artery Disease
Source: Int J Mol Sci. 2024 Aug 28;25(17):9304. doi: 10.3390/ijms25179304 (PMC11395148; doi:10.3390/ijms25179304)
Supplement: Supplementary file 1 [file ijms-25-09304-s001.zip › ijms-3147085-supplementary.pdf]

Supplementary Figure S1:

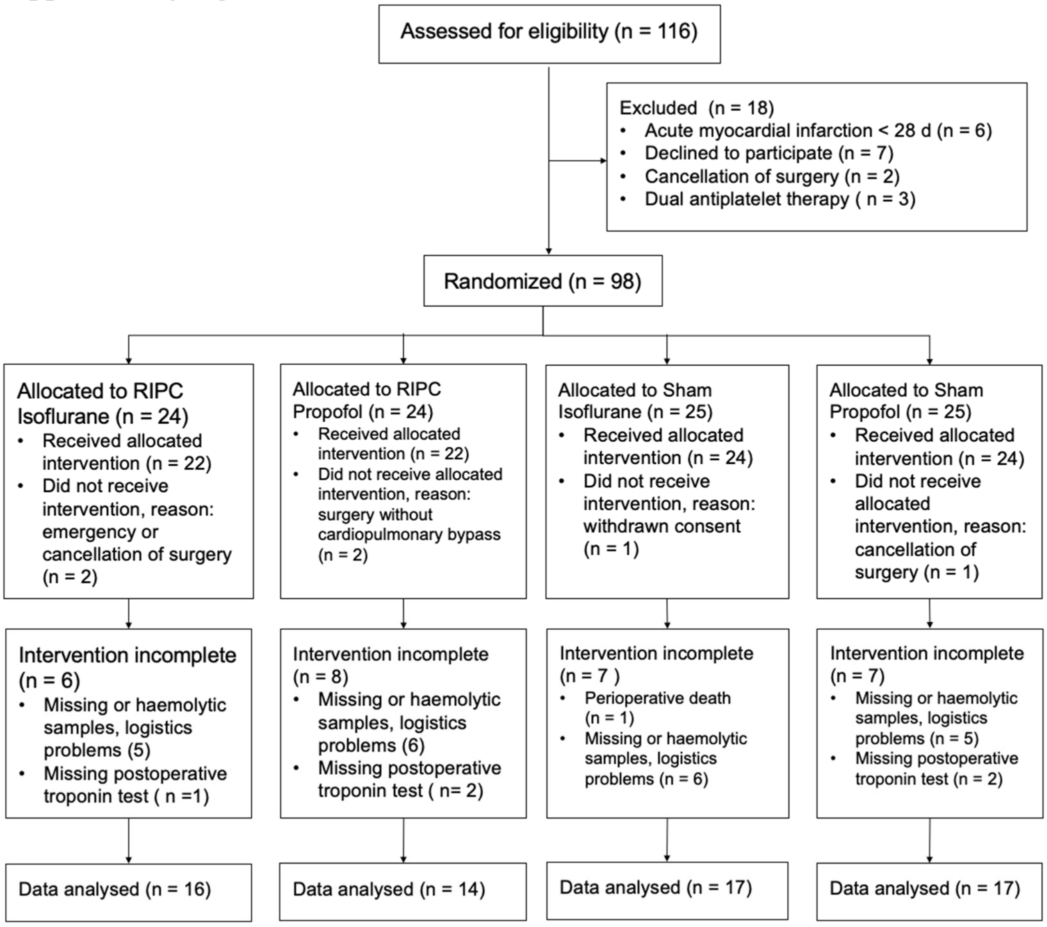

Supplementary Figure S1: CONSORT diagram. A total of 116 patients were screened, of which 98 were randomized. Finally 16 patients of the RIPC Isoflurane group, 14 patients of the RIPC Propofol group, 17 patients of the Sham Isoflurane group and 17 patients of the Sham Propofol group were included in the analysis.

Supplementary Figure S2:

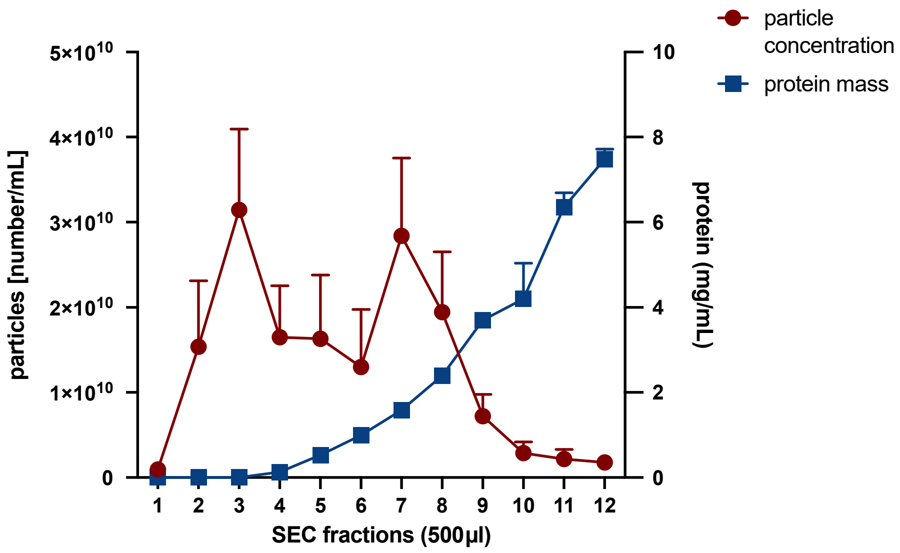

Supplementary Figure S2: SEC fractions: protein content (n = 1) and particle concentration (n = 4), fractions 1 to 12, EVs enriched with SEC protocol from plasma, statistics: Mean + SD.

Supplementary Figure S3:

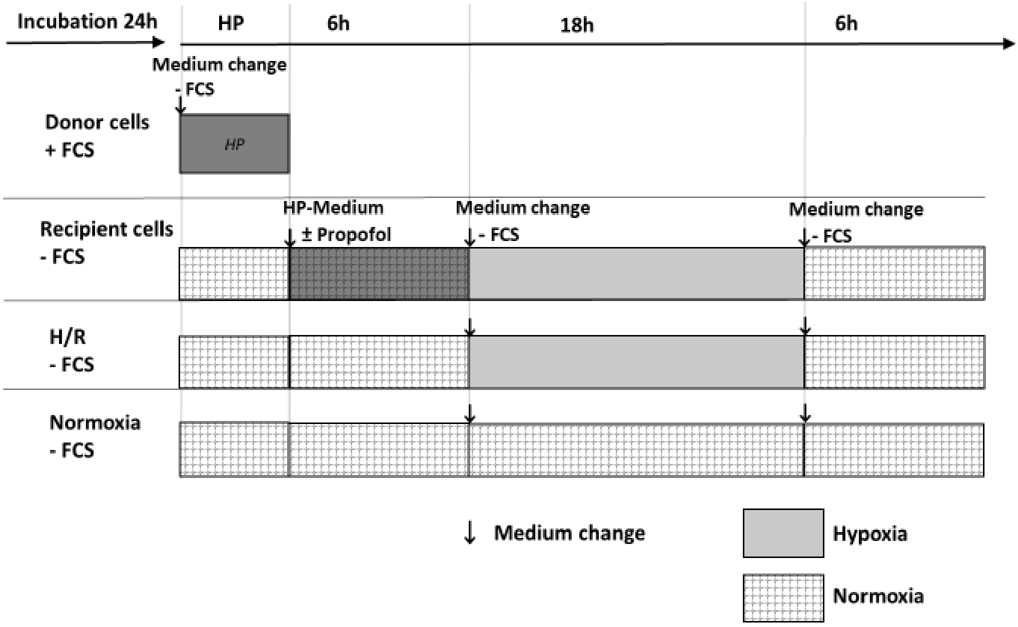

Supplementary Figure S3: Effect of preconditioning medium (Hypoxic preconditioning in vitro) on apoptosis of H9c2 rat cardiomyoblast: Experimental setup of simulated ischemia/reperfusion (SIR) with previous hypoxic preconditioning. Four conditions are represented in four lines. H9c2 rat cardiomyoblasts were subcultured in 6-well plates in DMEM medium (+ GlutaMAXTM + 4.5 g/L D-glucose,) ± 10% fetal calf serum (FCS) at 37 °C in an incubator for 24 hours. The medium of the donor cell group, which was subsequently subjected to hypoxic preconditioning (HP), was replaced with fresh DMEM medium (+ 4mM L-glutamine + 10mM Hepes buffer ± 10mM glucose, − FCS). The pH of these media was 7.4 in the glucose-containing medium (normoxia medium) and 6.5 in the medium without glucose (hypoxia medium). During HP, the remaining cell groups were incubated under normoxia (21% $O2$, 5% $CO2$). The HP medium was then transferred to the respective recipient cell group (±50 µM propofol) and incubated for 6 hours under normoxia. For subsequent simulated ischemia/reperfusion, the medium was renewed with normoxia medium in cells receiving normoxic treatment, or with hypoxia medium in cells receiving hypoxic treatment. The cells were then incubated for 18 hours under normoxia or hypoxia (1% $O2$, 5% $CO2$). This was followed by reperfusion, whereby the medium of all cells was replaced with fresh normoxia medium and incubation took place for six hours in a normoxic atmosphere. Flow cytometric apoptosis measurement was then performed in all groups.
